# GLI1 Confers Profound Phenotypic Changes upon LNCaP Prostate Cancer Cells That Include the Acquisition of a Hormone Independent State

**DOI:** 10.1371/journal.pone.0020271

**Published:** 2011-05-25

**Authors:** Sandeep K. Nadendla, Allon Hazan, Matt Ward, Lisa J. Harper, Karwan Moutasim, Lucia S. Bianchi, Mahmoud Naase, Lucy Ghali, Gareth J. Thomas, David M. Prowse, Michael P. Philpott, Graham W. Neill

**Affiliations:** 1 Centre for Cutaneous Research, Blizard Institute of Cell and Molecular Science, Barts and the London School of Medicine and Dentistry, Queen Mary University of London, London, United Kingdom; 2 Department of Biomedical Sciences, School of Health and Social Sciences, Middlesex University, Enfield, United Kingdom; 3 Cancer Sciences Division, University of Southampton School of Medicine, Southampton, United Kingdom; 4 Centre for Molecular Oncology and Imaging, Institute of Cancer, Barts and The London School of Medicine and Dentistry, Queen Mary University of London, London United Kingdom; East Carolina University, United States of America

## Abstract

The GLI (GLI1/GLI2) transcription factors have been implicated in the development and progression of prostate cancer although our understanding of how they actually contribute to the biology of these common tumours is limited. We observed that GLI reporter activity was higher in normal (PNT-2) and tumourigenic (DU145 and PC-3) androgen-independent cells compared to androgen-dependent LNCaP prostate cancer cells and, accordingly, GLI mRNA levels were also elevated. Ectopic expression of GLI1 or the constitutively active ΔNGLI2 mutant induced a distinct cobblestone-like morphology in LNCaP cells that, regarding the former, correlated with increased GLI2 as well as expression of the basal/stem-like markers CD44, β1-integrin, ΔNp63 and BMI1, and decreased expression of the luminal marker AR (androgen receptor). LNCaP-GLI1 cells were viable in the presence of the AR inhibitor bicalutamide and gene expression profiling revealed that the transcriptome of LNCaP-GLI1 cells was significantly closer to DU145 and PC-3 cells than to control LNCaP-pBP (empty vector) cells, as well as identifying LCN2/NGAL as a highly induced transcript which is associated with hormone independence in breast and prostate cancer. Functionally, LNCaP-GLI1 cells displayed greater clonal growth and were more invasive than control cells but they did not form colonies in soft agar or prostaspheres in suspension suggesting that they do not possess inherent stem cell properties. Moreover, targeted suppression of GLI1 or GLI2 with siRNA did not reverse the transformed phenotype of LNCaP-GLI1 cells nor did double GLI1/GLI2 knockdowns activate AR expression in DU145 or PC-3 cells. As such, early targeting of the GLI oncoproteins may hinder progression to a hormone independent state but a more detailed understanding of the mechanisms that maintain this phenotype is required to determine if their inhibition will enhance the efficacy of anti-hormonal therapy through the induction of a luminal phenotype and increased dependency upon AR function.

## Introduction

Prostate cancer (PCa) is the most common cancer in men and although tumours initially respond well to anti-hormonal treatment, the fact that many tumours acquire resistance to this form of therapy provides a major obstacle in treating advanced forms of the disease. Although the precise factors that initiate PCa remain unclear, numerous studies have described genetic lesions and aberrant signalling mechanisms that may contribute to tumour formation and progression, and those that help confer androgen independence are of particular interest as they may represent novel targets for therapeutic intervention (reviewed in [1]).

As with many tumour forms, the role of cancer stem cells (CSC) has received considerable attention in PCa biology, particularly with regard to tumour initiation but also progression and metastatic spread (reviewed in [2]). As prostate tumours display a predominantly luminal phenotype including AR expression, they are thought to derive from luminal secretory cells. However, based upon CD profiling and cytokeratin expression, basal-like characteristics have been identified in primary tumours and may be increased in metastatic and hormone-refractory tumours [3], [4]. Furthermore, basal/stem-like cells isolated from both primary tumours and cancer cell lines display greater tumourigenicity in mouse xenograft experiments [5], [6], [7], [8], [9], [10]. In contrast, Vander Griend et al [11] proposed that the cancer-initiating cell may be an intermediate AR-expressing cell that “acquires stem-like activity” and the heterogeneity of PCa is further highlighted by studies of mouse models: Wang et al [12] described a rare luminal stem cell population (expressing Nkx3-1) that can give rise to tumours whereas Lawson et al [13] found that basal epithelial stem cells were transformed more efficiently.

Hedgehog (HH) signalling represents a major developmental pathway that is implicated in the formation and progression of numerous tumour types including those of the skin, breast, pancreas, brain and lung. HH signalling, principally mediated by the downstream GLI (referring to both GLI1 and GLI2) transcription factors, is linked to tumourigenesis through the regulation of diverse mechanisms such as proliferation, differentiation, apoptosis, migration/invasion and the maintenance of CSC populations (reviewed in [14], [15], [16]).

Recent studies have described activation of HH signalling in PCa although the results have often been conflicting and the mechanism(s) by which GLI contribute to neoplasia are not well understood (reviewed in [17], [18]). For example, several studies have advocated that increased epithelial GLI1 expression promotes tumour formation [19], [20], [21]. In contrast, Fan et al [22] observed no significant difference in SHH or GLI1 mRNA levels between tumour and zone matched benign tissue and, more significantly, that GLI1 was expressed in the stromal, but not epithelial, component of BPH and PCa. Regarding the more advanced disease state, high levels of SHH protein and GLI1 mRNA have been described in metastatic samples and DHH, GLI1 and GLI2 have been linked with transformation to a hormone-refractory state [21], [23], [24], [25]. Moreover, recent studies have established a link between HH/GLI and AR signalling in the androgen-dependent (AD), luminal epithelial LNCaP prostate cancer cell line and demonstrated that GLI1 maintains cell viability in the absence of AR activity [25], [26], [27], [28].

Here we show that high GLI activity is observed in androgen-independent (AI) DU145 and PC-3 epithelial prostate cancer cell lines and that ectopic GLI1 promotes androgen independence in LNCaP cells which correlates with their transformation to a phenotype more characteristic of DU145 and PC-3 cells. However, GLI suppression does not promote an AD phenotype in DU145 or PC-3 cells. As such, early targeting of the GLI oncoproteins may impede progression to a hormone independent state, but this approach may not enhance the efficacy of anti-hormonal therapy in tumour cells that have lost AR expression and that are not dependent upon its signalling for their viability.

## Results

### Analysis of GLI expression in prostate cancer cells

**Figure 1:**
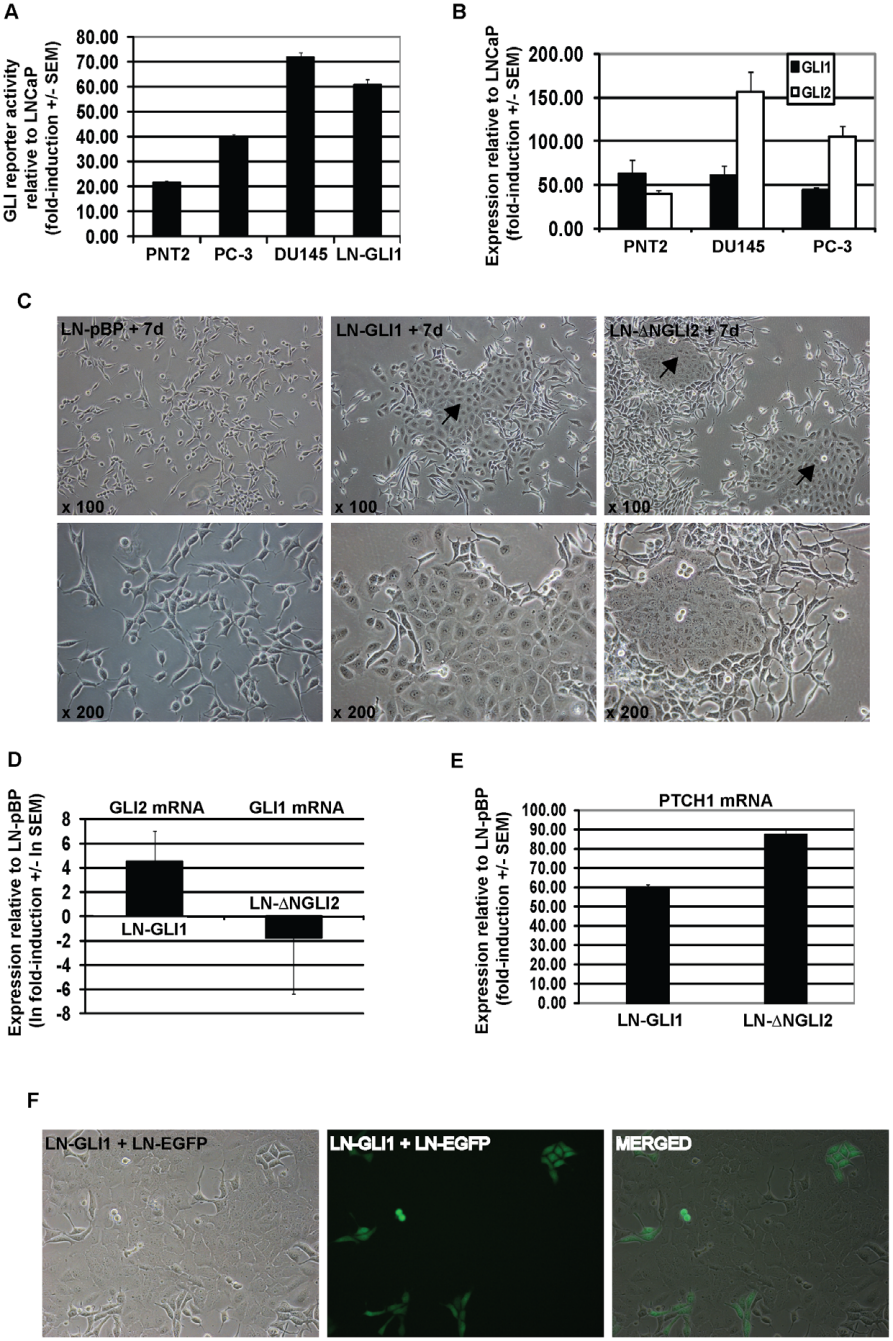
GLI activity is high in androgen-independent cell lines. (A) Analysis of GLI luciferase reporter activity in various androgen-independent cell lines and in comparison to the androgen-dependent LNCaP cell line. (B) Quantitative PCR analysis of GLI1 and GLI2 mRNA levels in the androgen-independent cell lines and in comparison to LNCaP cells (C) Cobblestone-like cells/colonies emerge in LNCaP cells with ectopic GLI1 or ΔNGLI2 expression (denoted by arrows). (D) qPCR analysis of PTCH1 mRNA expression in LNCaP-GLI1 and LNCaP-ΔNGLI2 cells. (E) qPCR analysis of GLI2 mRNA expression in LNCaP-GLI1 cells and GLI1 mRNA expression in LNCaP-ΔNGLI2 cells. (F) The morphology of LNCaP cells expressing EGFP does not change when co-cultured with LNCaP-GLI1 cells.

To investigate a putative role for GLI in prostate cancer, we first determined the level of GLI reporter activity in various prostate cell lines. GLI reporter activity was higher in the AI DU145 and PC-3 prostate cancer cell lines compared to the AD LNCaP prostate cancer cell line and reporter activity was also higher in the AI PNT-2 normal epithelial prostate cell line (Fig. 1A). Accordingly, GLI1 and GLI2 mRNA expression was higher in all AI cell lines compared to LNCaP cells (Fig. 1B). As such, we analysed the effect of over-expressing GLI1 and the active ΔNGLI2 mutant upon LNCaP cell biology. The most striking effect of ectopic GLI1 (eGLI1) and ΔNGLI2 related to cell morphology: in contrast to the characteristic spindle-like morphology of parental or control LNCaP-pBP (empty vector) cells, within a few days post-transduction cells/colonies with a cobblestone-like morphology were evident in LNCaP cells over-expressing eGLI1 or ΔNGLI2 (Fig. 1C). After drug selection, both LNCaP-GLI1 and LNCaP-ΔNGLI2 cells had completely transformed adopting a morphology reminiscent of PNT-2 or DU145 cells (refer Fig. 5A to view the fully transformed morphology). Ectopic GLI1 and ΔNGLI2 protein activity was confirmed by induction of PTCH1 mRNA (Fig. 1D). In addition, endogenous GLI2 mRNA was induced in LNCaP-GLI1 cells whereas, unexpectedly, endogenous GLI1 mRNA was suppressed in LNCaP-ΔNGLI2 cells revealing that the morphological change may be mediated by GLI2 (Fig. 1E). As DU145 and PC-3 cells express high levels of both GLI1 and GLI2 compared to LNCaP cells (Fig. 1B), we chose to further investigate the biology of LNCaP-GLI1 cells.

Initially, GLI reporter activity was measured in LNCaP-GLI1 cells and shown to be at a level comparable with PC-3 and DU145 cells (Fig. 1B, cf. columns 2–4). Subsequently, we addressed whether the ability of eGLI1 to induce the cobblestone-like morphology in LNCaP cells was through autonomous means or whether or not this required paracrine/juxtacrine signalling through molecules secreted by LNCaP-GLI1 cells. The morphology of LNCaP cells expressing EGFP did not change when co-cultured with LNCaP-GLI1 cells revealing that the cobblestone-like morphology is induced autonomously (Fig. 1F). However, we cannot discount the possibility that induction of the cobblestone-like morphology is mediated through receptors that are expressed in LNCaP-GLI1 cells (initially with a normal morphology) and that subsequently bind to molecules secreted by the same (or other) LNCaP-GLI1 cells acting through paracrine/juxtacrine signalling.

### GLI1 confers androgen-independence to LNCaP cells

**Figure 2:**
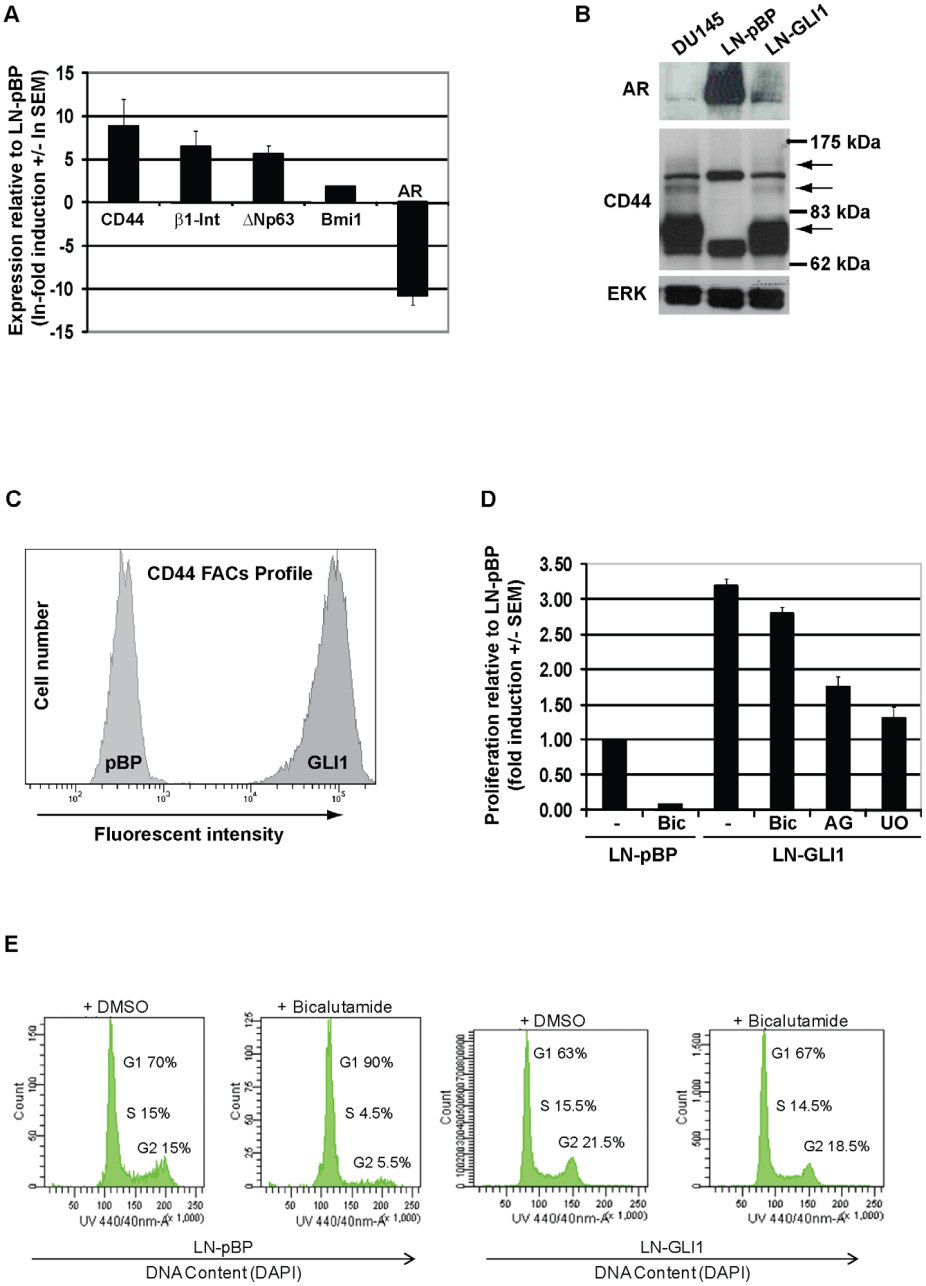
GLI1 induces an androgen-independent phenotype in LNCaP cells. (A) qPCR analysis of epithelial marker expression in LNCaP-GLI1 cells relative to LNCaP-pBP cells (n.b. the data is presented as natural logarithms so the relative induction of CD44 is almost 7000-fold). (B) Western blot analysis of AR and CD44 expression in LNCaP-pBP, LNCaP-GLI1 and DU145 cells (arrows denote CD44 isoforms common to LNCaP-GLI1 and DU145 cells). (C) FACS analysis of CD44 expression in LNCaP-pBP and LNCaP-GLI1 cells. (D) Proliferation assay to compare and to determine the effect of bicalutamide upon the proliferation rate of LNCaP-pBP and LNCaP-GLI1 cells as well as the effect of AG1478 (EGFR inhibitor) and U0126 (MEK inhibitor) upon the latter. (E) Analysis of the cell cycle by flow cytometry in LNCaP-pBP and LNCaP-GLI1 cells exposed to bicalutamide.

The expression of epithelial markers was investigated to determine if the luminal phenotype of LNCaP cells was altered by eGLI1: AR was strongly suppressed in LNCaP-GLI1 cells whereas the basal/stem-like markers CD44, β1-integrin, ΔNp63, and BMI1 were all increased (Fig. 2A); this was confirmed by Western blot analysis for AR and CD44, with increased cell surface expression of the latter confirmed by FACS (Figs. 2B and C). Due to the uniform global shift in CD44 expression we chose to employ the heterogenous population for further study. Regarding androgen dependence, whereas exposure to the AR inhibitor bicalutamide potently suppressed the proliferation of LNCaP-pBP cells, the increased proliferative potential of LNCaP-GLI1 cells was unaffected and this was verified by flow cytometry (Figs. 2D, lanes 1–4 and E). Therefore, as determined by epithelial marker expression and insensitivity to bicalutamide, these data suggest that eGLI1 induces regression (or de-differentiation) of LNCaP cells to a basal/stem-like form that is naturally independent of AR signalling for viability.

**Figure 3:**
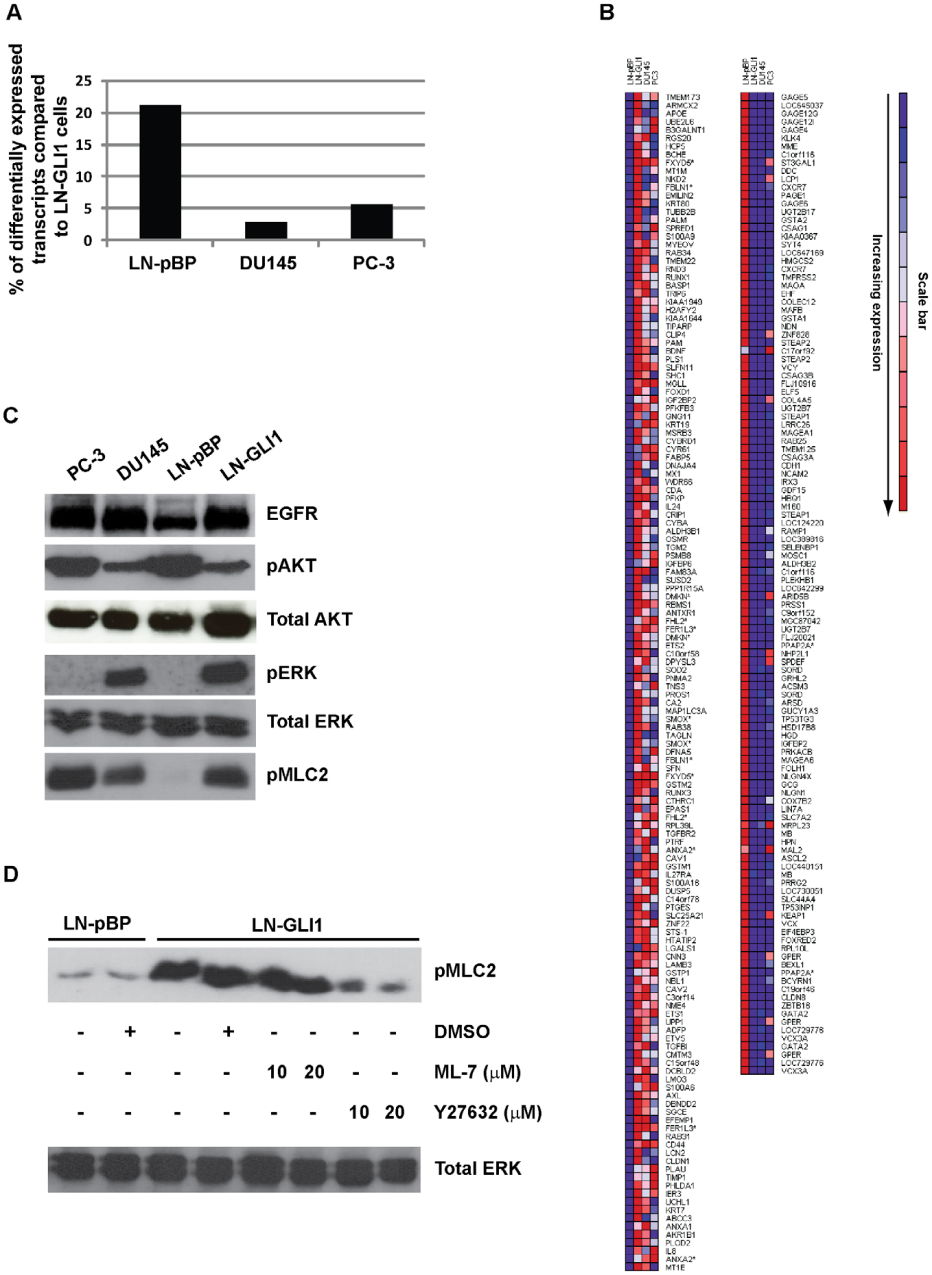
Ectopic GLI1 induces global changes in the gene expression profile of LNCaP cells. (A) A statistical comparison of global gene expression profiles to determine the percentage of transcripts that are expressed at significantly different levels in LNCaP-pBP, DU145 and PC-3 cells compared to LNCaP-GLI1 (Pearson correlation co-efficient ≥0.7, p<0.05) (B) Heat map denoting transcripts in LNCaP-GLI1 cells where the change in expression is both >10-fold and highly significantly different when compared to LNCaP-pBP cells (student's *t*-test, p<0.01): left panel lists increased genes, right panel lists decreased genes and DU145 and PC-3 cells are shown for comparison (* denotes transcript variants of the same gene). (C) Western blot analysis comparing the expression of certain signalling proteins between LNCaP-pBP and LNCaP-GLI1 cells with DU145 and PC-3 lysates included for comparison. (D) Phosphorylation of the cytoskeletal protein MLC2 is mediated by ROCK in LNCaP-GLI1 cells (n.b. the antibody for total MLC did not work in our hands).

To investigate this further, LNCaP-pBP, LNCaP-GLI1, DU145 and PC-3 cells were analysed by DNA microarrays: global array profiling revealed that the transcriptome of LNCaP-GLI1 cells was more similar to DU145 and PC-3 cells than to LNCaP-pBP cells thus revealing the extent to which LNCaP-GLI1 cells have changed phenotype (Fig. 3A). In direct comparison to LNCaP-pBP cells, the expression of 260 transcripts differed more than 10-fold (144 up and 116 down) in LNCaP-GLI1 cells (Fig. 3B and Figures S1 and S2). Functional classification of these transcripts produced 15 ontological groups including those associated with tumour biology such as cell-cell adhesion, cell motility, EMT (epithelial-mesenchymal transition) and hormone independence (Figure S3); the latter group including LCN2 (lipocalin 2) and CAV2 (caveolin 2) which were previously identified as part of a common signature for hormone independence in breast and prostate cancer [29]. The majority of the 144 increased transcripts were expressed at similar levels in LNCaP-GLI1 cells when compared to DU145 and/or PC-3 cells (<3-fold difference), whereas the expression of 12 transcripts (including LCN2) was >3-fold higher in LNCaP-GLI cells when compared to both cell types (Figure S1 and Table 1). Reciprocally, of the 116 decreased transcripts only one, MRPL23, was expressed >3-fold lower in LNCaP-GLI1 cells compared to both DU145 and PC-3 cells (Figure S2).

**Table 1:** Highly expressed transcripts in LNCaP-GLI1 cells.

Symbol	Accession No.	Fold change v LN-pBP	Fold change v DU145	Fold change v PC-3	Functional Group (Figure S2)
**ABCC3**	NM_003786.2	98.30	13.122	3.669	ATP and glucose metabolism
**CLDN1**	NM_021101.3	65.57	4.865	15.793	Cell-cell adhesion, EMT
**LCN2**	NM_005564.3	55.32	287.939	6.102	EMT, Hormone independence
**SMOX-4**	NM_175842.1	52.23	3.106	4.033	None
**TAGLN**	NM_003186.3	19.49	9.323	28.077	Cytoskeletal regulation
**SMOX-2**	NM_175840.1	19.30	3.047	3.529	None
**SUSD2**	NM_019601.3	15.83	19.827	10.819	None
**TUBB2B**	NM_178012.3	10.87	6.804	8.643	ATP and glucose metabolism, Rho GTPase signalling
**NKD2**	NM_033120.2	10.49	7.649	21.551	None
**HCP5**	NM_006674.2	10.33	3.221	5.446	None
**APOE**	NM_000041.2	10.04	8.952	4.633	Angiogenesis, Apoptosis regulation, Cytoskeletal regulation
**ARMCX2**	NM_177949.1	10.01	4.019	5.739	None

As well as DNA microarray profiling, the extent of major signalling pathway activation was assessed by Western blotting in LNCaP-GLI1 cells. Hormone independence is associated with EGFR pathway activation and although it has been established that EGFR mRNA expression is not greatly increased in AI cell lines ([29] and our microarray data), a strong increase in EGFR protein expression was observed in LNCaP-GLI1 cells to a level comparable with DU145 and PC-3 cells (Fig. 3C). ERK (Extracellular signal-Regulated Kinase) activity was also increased in LNCaP-GLI1 cells (Fig. 3C) and pharmacological inhibition of EGFR or ERK suppressed their high proliferative potential (Fig. 2D, cf. columns 1, 3, 5 and 6). Regarding AKT, although increased activity is associated with mutational inactivation of PTEN in LNCaP cells [30], [31], [32], eGLI1 reduced it to a level comparable with DU145 cells suggesting that there are mechanism(s) that could be exploited to obviate loss of this important tumour suppressor gene (Fig. 3C). Regarding the cytoskeleton, LNCaP-GLI1 cells displayed an increase of MLC2 (myosin light chain 2) phosphorylation that was similar to both DU145 and PC-3 cells (Fig. 3C and data not shown). MLC2 regulates the actin cytoskeleton (including stress fibre formation) and is itself regulated by MLCK (myosin light chain kinase) and ROCK (Rho-associated kinase); exposure to the ROCK inhibitor Y27632 but not the MLCK inhibitor ML-7 reduced MLC2 phosphorylation although this did not reverse the cobblestone-like morphology of LNCaP-GLI1 cells (Fig. 3D and unpublished observations). In summary, these data further demonstrate the extent to which LNCaP-GLI1 cells resemble DU145 and PC-3 cells.

### LNCaP-GLI1 cells do not display anchorage-independent growth

**Figure 4:**
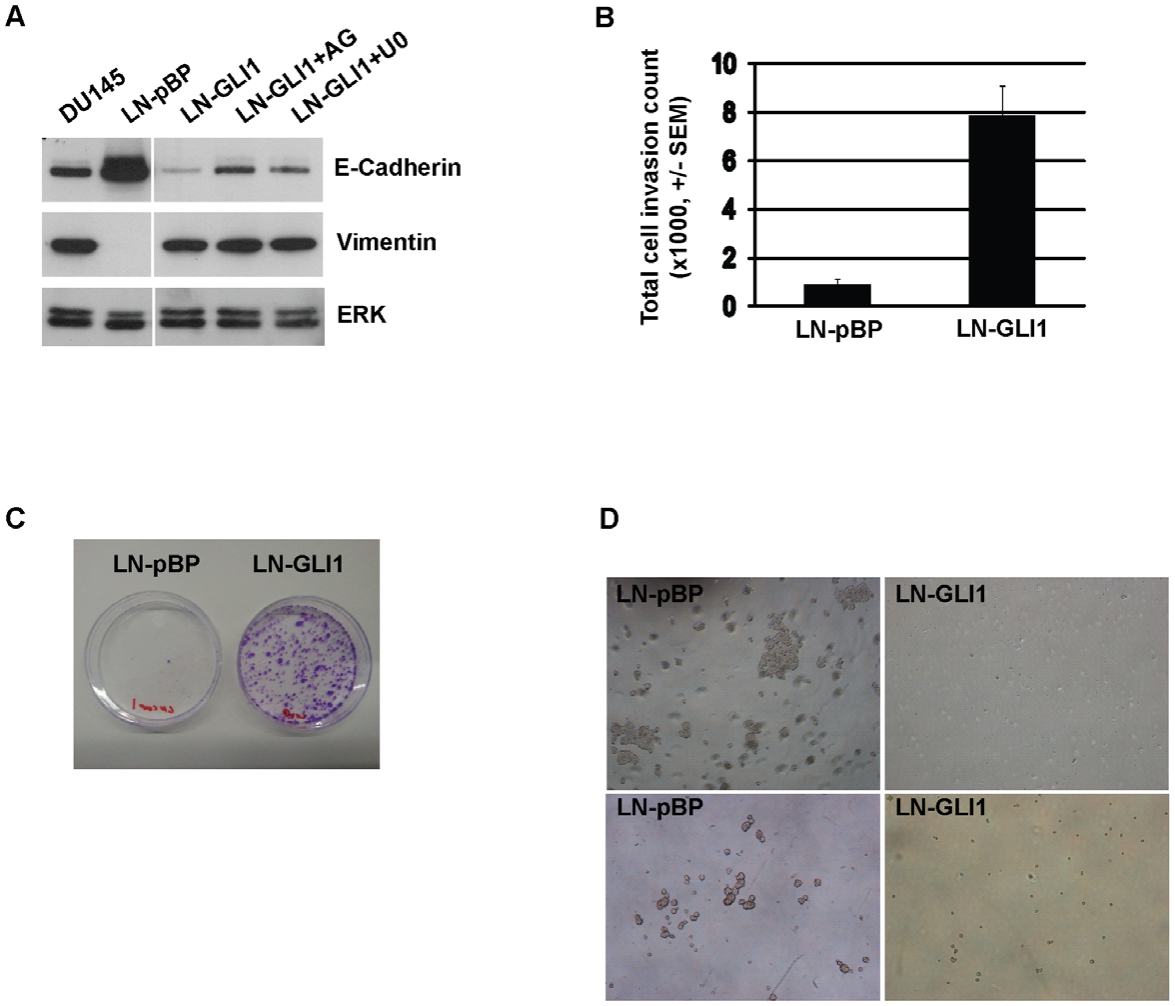
LNCaP-GLI1 cells display some stem-like characteristics. (A) Western blot analysis comparing expression of the EMT markers E-cadherin and vimentin between LNCaP-GLI1 and LNCaP-pBP cells (n.b. the decrease of E-cadherin in LNCaP-GLI1 cells is partially reversed in the presence of the EGFR inhibitor AG1478 and to a lesser extent the MEK inhibitor U0126). (B) Transwell invasion assay comparing the invasive potential of LNCaP-pBP and LNCaP-GLI1 cells through a Matrigel substrate. (C) Clonogenicity assay assessing the colony-forming ability of LNCaP-pBP and LNCaP-GLI1 cells when seeded at low density. (D) Anchorage-independent growth is observed in LNCaP-pBP cells but not LNCaP-GLI1 cells (top panel - soft agar colony assay; bottom panel - prostasphere assay).

HH/GLI signalling regulates normal and cancer stem cell populations and recent studies have described how EMT is an inherent trait of such cells [15], [16], [33]. Interestingly, despite their cobblestone-like morphology, the results of the microarray revealed that eGLI1 induces EMT in LNCaP cells (Figure S3). Indeed, decreased E-Cadherin and increased vimentin expression was confirmed by Western blotting, although this was not dependent upon EGFR or MEK-ERK signalling [34] (Fig. 4A). Accordingly, LNCaP-GLI1 cells were highly invasive through a Matrigel™ substrate (Fig. 4B) and they also displayed greater clonal growth when seeded at low density (Fig. 4C). However, despite the expression of ‘stemness’ markers (including CD44, β1-integrin and BMI1), EMT and greater clonal growth (Figs. 2A, 4A and 4C), unlike control cells LNCaP-GLI1 cells did not form prostaspheres in suspension or colonies in soft agar (Fig. 4D). To address the possibility that LNCaP-GLI1 cells do not proliferate in 3-D culture because they are not able to differentiate towards a luminal phenotype (i.e. because of constitutive eGLI1 expression), DU145 cells were also cultured under the same conditions. No colonies were observed in either assay with DU145 cells suggesting that AR^−^ cells are poorly clonogenic in anchorage-independent *in vitro* culture systems (data not shown); this is supported by Thiyagarajan et al [35] who observed that DU145 (and PC-3 cells) were much less proliferative in soft agar compared to LNCaP cells although some colony growth was evident in their study.

### GLI suppression does not promote a luminal-like phenotype in androgen-independent prostate cancer cells

**Figure 5:**
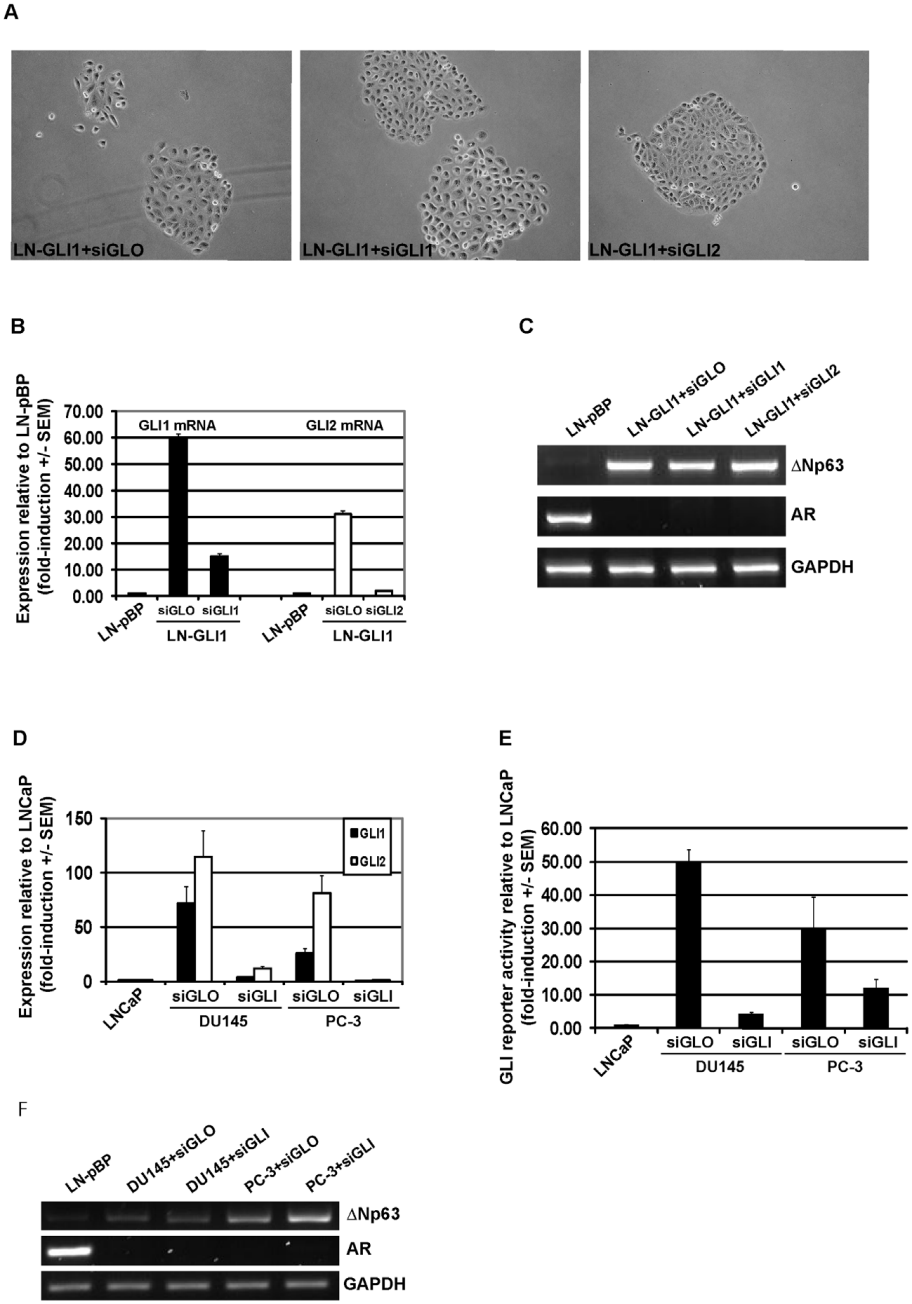
GLI suppression does not induce a luminal-like phenotype in androgen-independent cells. (A) The transformed morphology of LNCaP-GLI1 cells does not reverse upon transfection with GLI1 or GLI2 siRNA. (B) qPCR analysis of GLI1 and GLI2 mRNA in LNCaP-GLI1 cells transfected with GLI1 or GLI2 siRNA. (C) RT-PCR analysis of ΔNp63 and AR mRNA in LNCaP-GLI1 cells transfected with GLI1 or GLI2 siRNA. (D) qPCR analysis of GLI1 and GLI2 mRNA in DU145 and PC-3 cells transfected with GLI1 and GLI2 siRNA. (E) GLI reporter activity is suppressed in DU145 and PC-3 cells transfected with GLI1 and GLI2 siRNA (n.b. reporter activity may be influenced by GLI3 expression in PC-3 cells [17]). (F) RT-PCR analysis of ΔNp63 and AR mRNA in DU145 and PC-3 cells transfected with GLI1 and GLI2 siRNA.

Finally, we sought to determine if targeted suppression of GLI was sufficient to reverse the transformed phenotype of LNCaP-GLI1 cells or to induce a luminal-like phenotype in DU145 or PC-3 cells. Transfection of LNCaP-GLI1 cells with GLI1 or GLI2 siRNA did not influence the morphology of LNCaP-GLI1 cells nor was there any change in the expression of ΔNp63 or AR mRNA (Figs. 5A–C and Figure S4A); this indicates that the phenotypic conversion induced by eGLI1 in LNCaP cells is irreversible and that maintenance of the AI phenotype is not dependent upon GLI2. Regarding DU145 and PC-3 cells, the efficacy of double GLI1/GLI2 knockdowns was confirmed by a decrease of GLI reporter activity but there was no change in cell morphology nor was there any change in the expression of ΔNp63 or AR mRNA (Figs. 5D–F, Figure S4B and unpublished observations). We also employed the GLI inhibitor GANT61 (30 µM) [36] but this was less efficient at suppressing GLI reporter activity than RNAi (data not shown). As such, although AI prostate cancer cells display high GLI mRNA expression and activity and eGLI1 is able to promote an AI phenotype in LNCaP cells, GLI suppression does not promote a luminal-like and AD phenotype in AI prostate cancer cells.

## Discussion

The role of HH signalling has proven contentious in PCa biology; this includes debate as to whether or not the pathway contributes to primary tumour formation as well the actual mode of signalling (autocrine or paracrine). In addition, there has been conflicting data as to whether or not GLI expression is mediated through canonical or non-canonical pathways in PCa cell lines (reviewed in [18]). We have not addressed the nature of GLI regulation but have shown that the AI cell lines PNT-2, DU145 and PC-3 display higher levels of GLI mRNA than the AD LNCaP prostate cancer cell line and this correlates with increased GLI reporter activity (Figs. 1A and B). The fact that GLI1 expression was comparable between normal PNT-2 cells and tumourigenic DU145 and PC-3 cells was unexpected but in contrast to Karhadkar et al [21], we also found that GLI1 mRNA was strongly expressed in commercial primary prostate basal epithelial cells (PrECs), though a faithful comparison to the cell lines used in this study was not possible because PrECs are cultured in specialist medium that does not contain serum (S.K.N. and G.W.N., unpublished). Despite these observations, at the protein level GLI1 is rarely detected in the basal layer of normal human prostate tissue whereas expression is more prevalent in hyperplastic basal cells and carcinomas [20]. As such, in a manner akin to GLI2 regulation [37], although GLI1 mRNA expression is constant between normal and tumourigenic cells, the protein may be stabilised in the latter (possibly through Fused [38]) and this, along with the GLI2, could account for the increase in GLI reporter activity.

Our data suggests that GLI1 induces androgen-independence in LNCaP cells through its ability to induce a basal-like phenotype that is associated with basal cell populations and that is naturally independent of AR activity; this is supported by reduced AR expression combined with an increase of numerous basal/stem-like markers. Chen et al [26] also described a role for GLI1 in promoting AI growth in LNCaP cells but this was not associated with reduced AR expression and may reflect the fact that eGLI1 expression was lower in their system as determined by a lesser fold-increase of GLI1 reporter activity. Although our studies were performed on a heterogenous cell population, the phenotype was uniform and we have not been able to isolate LNCaP-GLI1 clonal lines that maintain normal LNCaP morphology indicating that retroviral eGLI1 promotes an ‘all or nothing’ response, but as the level of GLI reporter activity was comparable with DU145 and PC-3 cells this indicates that our system has biological relevance. How eGLI1 mediates the transformation of LNCaP cells has not been elucidated but may involve multiple mechanisms: eGLI1 inhibition of AR signalling alone is unlikely to initiate the phenotypic change but, combined with its ability to sustain cell viability in the absence of AR signalling [27], [28], this may compound the effects of its principal role as a transcriptional activator.

As noted above, eGLI1 increased total GLI activity in LNCaP cells to a level comparable with DU145 and PC-3 cells. Microarray profiling revealed that the transcriptome of LNCaP-GLI1 cells was similar to both DU145 and PC-3 cells with the expression of certain genes comparable to one or both cell lines. This probably reflects the genotype of each cell and the fact that GLI activity and target gene activation are influenced by signalling enzymes (including ERK and AKT) that are differentially activated in each cell type [39], [40], [41]. Intriguingly, Nadiminty et al [42] recently listed a set of 50 target genes induced by NF-κB2 in LNCaP cells, 15 of which are present in our list of 144 genes induced >10-fold by eGLI1 in LNCaP cells (including LCN2) suggesting that NF-κB2 activation is one of the mechanisms through which eGLI1 elicits its effect in LNCaP cells (Figure S1, transcripts highlighted in red).

Regarding the expression of transcripts that are highest in LNCaP-GLI1 cells (Table 1), ABCC3 is of particular interest because it encodes a protein that belongs to the ABC (ATP-Binding Cassette) family of transporters that confer drug resistance and that are highly expressed in normal and cancer stem cells (reviewed in [43]). HH/GLI1 signalling has been shown to regulate the expression of Pgp (ABCB1) and BCRP (ABCG2) in various cancer cell lines including PC-3 [44]. In addition, the SMO inhibitor GDC-0449 was recently shown to inhibit the drug resistance properties of Pgp and and BCRP [45]. The shuttle/transport protein lipocalin 2 (LCN2) is also of particular interest: lipocalin 2 was identified as one half of a complex with matrix metalloproteinase MMP-9 that is elevated in the urine of cancer patients (notably breast, bladder, pancreas and prostate) [46], [47], and it also forms part of a common gene signature for hormone independence in breast and prostate cancer [29]. Functionally, lipocalin 2 protects MMP-9 from degradation and recently it has been shown to promote EMT by modulating ERα (oestrogen receptor alpha) and SLUG expression in MCF-7 cells. In addition, lipocalin 2 negates the response of MCF-7 cells to oestrogenic stimulation [48]. GLI1 also represses ERα in MCF-7 cells and negates their response to oestrogenic stimulation as well as promoting hormone independence [49]. These studies provide evidence for functional overlap between GLI1 and lipocalin 2 in breast cancer and, accordingly, the expression of both proteins is associated with the ER^−^ phenotype [49], [50], [51], [52]. Similarly, although the tight junction protein claudin 1 (CLDN1) is often decreased in breast tumours [53], [54], [55], high expression has been described in ER^−^ tumours [56], [57]. In the prostate, claudin1 expression is high in the basal layer of benign tissue and its expression decreases with increasing tumour aggressiveness [58], [59]. A similar pattern of expression has also been described for the actin-binding protein transgelin (TGLN) [60]; although this may appear anomalous, it is feasible that these proteins are expressed at high levels in a small population of basal-like CSCs that are not easily detected by immunohistochemistry in tumours that display a predominantly luminal (AR^+^) phenotype. Indeed, transgelin is more highly expressed in the CD44^+^ fraction of DU145 and LNCaP cells [61] and some evidence of increased HH signalling has been described in an invasive subpopulation of DU145 cells that express higher levels of CD44 as well as the stem cell marker NANOG [62].

Although HH/GLI1 signalling modulates CSC biology in various tissues, defining its role in PCa is complicated by the fact that cancer-initiating cells may stem from AR^−^ (basal) or AR^+^ (intermediate/luminal) populations [5], [6], [7], [8], [11], [12], [13]. If PCa arises from basal/stem-like cells then based upon the results presented here, theoretically they would express high GLI levels. Conversely, if PCa arises from luminal (or intermediate) cells that express AR then they would be expected to express low or absent levels of GLI. This study has not addressed the role of GLI in tumour initiation but its expression is increased in hyperplastic basal cells that co-express CD44 and p63 [20]. Interestingly, the same authors demonstrated GLI expression in localised prostate cancer; this may be unexpected as primary tumours are considered to display a predominantly luminal phenotype (i.e. p63^−^/AR^+^) but this probably reflects lower GLI activity compared to more aggressive tumours. However, a meta-analyses of microarray datasets has shown that a considerable number of localised prostate tumours display a gene expression profile which is indicative of hormone-independence and reduced AR expression [29]. Indeed, it would be interesting to determine if GLI expression was evident in these datasets although they may have been subject to the same technical limitations that are discussed at the end.

Less equivocal is the role of GLI in advanced PCa: high levels of GLI1 mRNA have been described in metastatic tumours and both GLI1 and GLI2 have been linked with androgen-independence [21], [23], [24], [25], [27]. The basal cytokeratin K5 is expressed in metastatic tumours and this is increased in tumours subject to androgen deprivation as well as those that are hormone-refractory [4]. Moreover, CD profiling and expression studies have shown that basal cells are present in advanced/metastatic tumours [3], [63], [64]. Intriguingly, Liu et al [63] identified the EMT marker vimentin as part of a basal cDNA signature in metastatic prostate tumours. Combined with the fact that EMT is synonymous with CSC biology [33] and that prostate stem/progenitor cells often express basal markers (reviewed in [2]), this suggests that there is synergy between EMT and the basal phenotype in prostate CSC biology and these phenomena may be linked through HH/GLI signalling.

Regarding the mechanisms that control GLI expression in advanced PCa, as well as canonical HH signalling [25], GLI may be regulated by TGF-β (via Smad3) [65]. Inhibition of TGF-β or Smad3 has been shown to suppresses the growth and metastasis of AI tumours in Nude mice (but not tumour incidence) and, as for GLI, Smad3 is expressed at considerably higher levels in DU145 cells compared to LNCaP cells [66], [67]. Therefore, TGF-β/Smad3 signalling may, in part, account for increased GLI expression in advanced PCa and this also correlates with the fact that TGF-β is associated with EMT and CSC biology [33]. Based upon the fact that GLI reporter activity was high in DU145 and PC-3 cells and that eGLI1 induced an AI phenotype in LNCaP cells, we had surmised that GLI inhibition may induce an AD phenotype in DU145 and PC-3 cells through increased AR expression. Surprisingly, neither eGLI1 nor GLI2 suppression reversed the phenotype of LNCaP-GLI1 cells; although we cannot discount the possibility that protein expression was not sufficiently suppressed, this suggests that the transformation is irreversible or that once the process has occurred it is no longer dependent upon GLI activity and this is supported by the fact that GLI suppression did not influence the phenotype of DU145 or PC-3 cells as determined by marker gene expression (Fig. 5F). A global screening approach may be required to determine if it is possible for DU145 or PC-3 cells to trans-differentiate towards a luminal phenotype that is dependent upon AR function but this may not be possible for the former as loss of AR expression is associated with promoter methylation (Sasaki et al, 1992). However, this approach may be viable for PC-3 cells as well as those hormone refractory tumours where loss of (or reduced) AR expression is not associated with promoter methylation [68], [69]. MicroRNAs provide an attractive target for further investigation as they can regulate multiple genes, including AR, and are associated with stem cell biology, tumour biology and hormone independence [70], [71], [72], [73], [74], [75]. This will be supported by delineating the mechanisms through which the GLI oncoproteins promote hormone independence and as these may be common to the pathogenesis of breast and prostate cancer such investigations are clearly warranted. Moreover, the fact that GLI inhibition has been shown to negatively influence the proliferation and clonogenic/tumourigenic potential of prostate cancer cell lines as well as increasing their sensitivity to cancer drugs enhances their attractiveness as target proteins for therapeutic intervention [19], [23], [35].

Finally, in this study we found that the microarray failed to detect GLI1 or GLI2 as highly expressed transcripts in LNCaP-GLI1, DU145 or PC-3 cells. Indeed, from the normalised data the expression of GLI1 was constant between all four cell lines analysed and GLI2 was only slightly increased in LNCaP-GLI1 cells (2.24-fold), DU145 cells (2.95-fold) and PC-3 cells (2.71-fold) which does not correlate with the qPCR data (Fig. 1B). The GLI1 probe sequence corresponds to a region within the last exon of GLI1 (NM_005269.2) and should detect both eGLI1 and endogenous GLI1 in all cell lines. In addition, the lack of signal is unlikely to be due to the presence of GLI1 splice variants as these are N-terminal [76], [77]. Regarding GLI2, the probe sequence corresponds to the non-coding region of the last exon (NM_005270.4) and should also detect the known splice variants [78], [79], [80]. As such, failure to capture GLI1 or GLI2 mRNA appears to be a technical issue and it is likely that the expression level of these genes has been misrepresented in other datasets generated with the Illumina platform.

## Materials and Methods

### Vector construction

Human GLI1 encoding cDNA was amplified by standard PCR with Pfu Turbo DNA Polymerase (Stratagene) and pBluescript-GLI1 (a gift from Kenneth Kinzler) as the template: the primers contained 5′ phosphate groups (Forward, 5′-CTCTGAGACGCCATGTTCA-3′ and Reverse, 5′-GATTCCCTACTCTTTAGGCA-3′). The amplicon was cloned into pBabePuro blunted at the Sal1 site to create pBP-GLI1; the integrity of the coding region was verified by sequencing. ΔNGLI2β coding cDNA was isolated from pcDNA4/TO-HisΔNGLI2β (Regl et al, Oncogene 2004) by Pme1 digestion and cloned into pBabePuro blunted at the Sal1 site to create pBP-ΔNGLI2β. The ΔNGLI2β mutant is lacking the first 328 amino acids and is highly transcriptionally active compared to the wild-type GLI2β protein [79].

### Cell culture and retroviral transduction

The prostate cancer cell lines LNCaP, DU145 and PC-3 were obtained from the European Collection of Cell Cultures (through Sigma-Aldrich) and normal prostate epithelial PNT2 cells were kindly provided by Norman Maitland (University of York) [81]. All cells were maintained in RPMI 1640 medium supplemented with 10% FBS, L-Glutamine (2 mM), penicillin (50 U/ml) and streptomycin (50 µg/ml) (all Lonza). Amphotropic retroviral particles harbouring pBabePuro (empty vector), pBP-GLI1 or pBP-ΔNGLI2 were created as described previously [82] using the Phoenix packaging cell line obtained from the Nolan Laboratory (http://www.stanford.edu/group/nolan/retroviral_systems/phx.html). To create the LNCaP-pBP, LNCaP-GLI1 and LNCaP-ΔNGLI2 stable cell lines, parental LNCaP cells were exposed to the corresponding viral particles in the presence of polybrene (5 µg/ml) and centrifuged at 300×g for 1 hr at 32°C. Subsequently, the cells were allowed to recover for 72 hrs prior to selection with puromycin (1 µg/ml) for up to 1 week and beyond the time when all the control (non-transduced) cells had expired.

### Reporter assay

Cells were seeded at a density of 2,000 cells/cm^2^ in triplicate (6-well plates) and transfected 48 hr post-seeding with 1 µg of the GLI firefly luciferase reporter pGL3-6GBS [83] and 1 µg of a pCMV-Renilla normalisation vector using 3 µl of Fugene6 (Roche). Cells were harvested 24 hr post-transfection and analysed for luciferase activity using the Dual Luciferase Assay Kit (Promega) and a FLUOStar OPTIMA reader (BMG Labtech) (n = 3).

### Proliferation and clonogenicity assays

LNCaP-pBP and LNCaP-GLI1 cells were seeded at a density of 500 cells/cm^2^ and exposed to bicalutamide (10 µg/ml), AG1478 (1 µM), UO126 (5 µM) or vehicle (DMSO) 24 hr post-seeding. Fresh drug/media was added after another 72 hr and the cells were trypsinised and counted 7 days post-seeding using a Casy 1 counter (Sharfe System GmBH) (n = 3). For clonal growth, LNCaP-pBP and LNCaP-GLI1 cells were seeded at a density of 50 cells/cm^2^ in triplicate and cultured for 10 days prior to fixing in 3% paraformaldehyde and staining with crystal violet (n = 3).

### Western Blotting

Protein lysates were prepared as described previously [82] with separation and transfer to nitrocellulose membrane performed according to standard protocols. In summary, cells were seeded at a density of 7000/cm^2^ and harvested 72 hr post-seeding: where indicated pharmacological agents including AG1478 (1 µM), UO126 (5 µM), ML-7 (10–20 µM) and Y27632 (10–20 µM) were added 24 hr before harvesting. Primary antibodies used were: CD44 (eBiosciences); GLI1 C-18 and EGFR SC-03 (Santa Cruz Biotechnology); AR, E-cadherin and vimentin (BD Biosciences); ERK (also used as a loading control), phospho-ERK (E10), AKT, phospho-AKT (Ser473) and phospho-MLC2 (Cell Signalling Technology). Secondary HRP-linked antibodies were obtained commercially (DAKO) and immunodetection performed with ECL+ reagent (GE Healthcare).

### Quantitative polymerase chain reaction

Total RNA was isolated using the RNeasy Plus Mini Kit (Qiagen, Valencia, CA) with 3 µg of RNA used to prepare 30 µl of cDNA using the Superscript® First Strand Synthesis System (Invitrogen Life Science). Quantitative polymerase chain reactions (qPCR) were performed with Platinum™ SYBR® Green qPCR Supermix (Invitrogen Life Science) and analysed on a Corbett Rotor-Gene 3000. The melting curve graph of the PCR product indicated that the data generated was from a single product and confirmed by running on a 1% agarose gel. Relative induction values (x) were calculated using the formula x = 2^−ΔΔCT^ where Ct represents the mean threshold cycle of replicate analyses, ΔCt represents the difference between the Ct values of the target gene and the reference gene GAPDH, and ΔΔCt is the difference between the ΔCt values of the target gene for each sample compared to the ΔCt mean of the reference sample (LNCaP or LNCaP-pBP). Primers used were 5′-3′: GLI1 F-GAAGACCTCTCCAGCTTGGA, R-GGCTGACAGTATAGGCAGAG; GLI2 F-GGGTCAACCAGGTGTCCA, R-GATGGAGGGCAGGGTCAAGGA; PTCH1 F- ACTCGCCAGAAGATTGGAGA, R- TCCAATTTCCACTGCCTGTT; CD44 F-GTGATCAACAGTGGCAATGG, R-CCACATTCTGCAGGTTCCTT; β1-Int F-GGGGTAATTTGTCCCGACTT, CATCTGCGAGTGTGGTGTCT; ΔNp63 F- GTCCCAGAGCACACAGACA, R- GAGGAGCCGTTCTGAATCTG; Bmi1 F- CCAGGGCTTTTCAAAAATGA, R-CCGATCCAATCTGTTCTGGT; AR F- TACCAGCTCACCAAGCTCCT, R-GCTTCACTGGGTGTGGAAAT; PSA F-CACAGCCTGTTTCATCCTGA, R-AGGTCCATGACCTTCACAGC and GAPDH F-GCCTTCCGTGTCCCCACTGC, R-GCTCTTGCTGGGGCTGGTGG.

### Flow cytometry

For cell cycle analysis, 4000 cells/cm^2^ were seeded in a T-25 flask and exposed to bicalutamide (10 µg/ml) or vehicle (DMSO) for the final 48 hrs before harvesting (96 hrs post-seeding). Trypsinised cells were washed twice at 1200 RPM for 5 min in PBS with the pellet then fixed in cold sterile 70% ethanol before storing at 4°C overnight. Fixed cells were then washed ×3 at 1200 RPM for 5 min in 5 ml PBS. During the third wash 100 µl of cells from one of the cell lines was aliquoted separately to calibrate the FACS machine. After washing, the pellet was re-suspended in 300 µl of DAPI solution (10 µls of 0.1 mg/ml DAPI, 25 µls of 5.0 mg/ml RNase-A, 380 µls of 100 mM sodium citrate in 485 µls PBS) and incubated in the dark for 30 min at RT. DAPI-labelled cells were loaded on a BD FACS machine (LS-RII) and analysed with DIVA software.

For FACS, cells were incubated with 10 ml of versene for 15 min at 37°C, neutralised with RPMI/10% FCS then centrifuged at 1200 RPM for 5 min at RT. The cell pellet was washed twice in PBS then incubated for 1 hr in the dark with fluorescently-labelled CD44 antibody (14-0441, eBioscience) diluted 1∶500 in PBS. CD44-labelled cells were loaded on a BD FACS machine (LS-RII) and analysed with DIVA software.

### Gene expression and statistical analyses

Gene expression profiling was performed using a HumanHT-12v4 BeadChip read by the HiScanSQ system (Illumina, Inc). All samples were analysed in triplicate and the results were normalised to the LNCaP-pBP transcriptome using Bead-Studio® software (Illumina, Inc): the raw data has been deposited with GEO (Accession No.: GSE27231) and is MIAME compliant. Normalised data was filtered for significant genes (student's *t*-test; p<0.01) with a >10-fold expression difference (±) using custom designed software plugged in to Excel. Significant genes were grouped using DAVID 6.7 software [84], [85] and further verified by consensus clustering using GenePattern software [86]. A direct global array comparison of the LNCaP-GLI1 transcriptome versus the LNCaP-pBP, DU145 and PC-3 transcriptomes was done using the Pearson correlation matrix (p<0.05) using MeV v.4.5.1 software (TM4, Microarray Software Suite) [87], [88].

### Transwell invasion and anchorage-independent assays

Cell invasion assays were performed over 72 hr using Matrigel-coated (diluted 1∶2 with RPMI 1640) polycarbonate filters (Transwell, BD Biosciences). Cells (50,000 seeded) invading the lower chamber were trypsinised and counted using a Casy 1 counter (Sharfe System GmBH) (n = 6). For soft agar growth, 2500 cells/ml were re-suspended in 0.4% agarose on a 1% agarose bed (diluted in RPMI 1640/10% FCS) and cultured for up to 3 weeks with medium covering the top layer being replaced every 3–4 days (n = 3). For prostasphere growth, 500 cells/ml were re-suspended in DMEM/F12 medium supplemented with B27 and N2 (Invitrogen) in non-adherent plates and cultured for up to 3 weeks (n = 3).

### RNA interference

7000 cells/cm^2^ were reverse-transfected with control siGLO (Dharmacon) or siRNA targeting GLI1 (Ambion *Silencer*® Select s5816) and/or GLI2 (Ambion *Silencer*® Select s5817) using the Hiperfect (Qiagen) transfection reagent to produce a final concentration of 30 nM; fresh medium was added 24 hr post-seeding. RNA was isolated 96 hr post-seeding or cells were transfected with pGL3-6GBS and pCMV-Renilla 72 hr post-seeding prior to harvesting for luciferase activity 96 hr post-seeding (n = 3).

## Supporting Information

Figure S1 **Excel worksheet with the raw expression data of the positively regulated genes presented within the left heat map of** **Fig. 3B** **.** The transcripts additionally presented in Table 1 are underlined and those that were identified as targets of NF-κB2 [41] (see Discussion) are highlighted in red.
(TIF)

Figure S2 **Excel worksheet with the raw expression data of the negatively regulated genes presented within the right heat map of** **Fig. 3B** **.**
(TIF)

Figure S3 **Mini heat maps denoting functional groups of the genes presented in** **Fig. 3B** **and Figures S1 and S2.**
(TIF)

Figure S4 **qPCR analysis of ΔNp63 mRNA expression in LNCaP-GLI1, DU145 and PC-3 cells.**
(TIF)
