# Supplementary figures and images for: GLI1 Confers Profound Phenotypic Changes upon LNCaP Prostate Cancer Cells That Include the Acquisition of a Hormone Independent State
Source: PLoS One. 2011 May 25;6(5):e20271. doi: 10.1371/journal.pone.0020271 (PMC3102098; doi:10.1371/journal.pone.0020271)

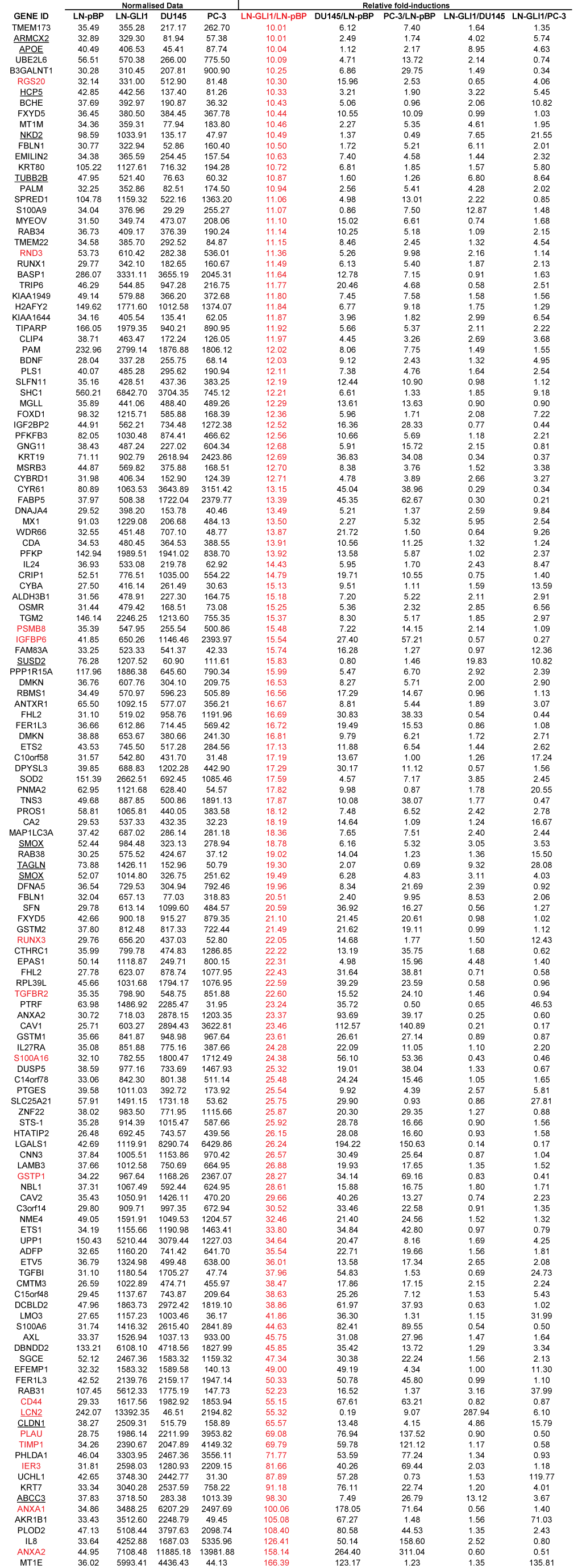

Supplement: Figure S1 — Excel worksheet with the raw expression data of the positively regulated genes presented within the left heat map of Fig. 3B . The transcripts additionally presented in Table 1 are underlined and those that were identified as targets of NF-κB2 [41] (see Discussion) are highlighted in red. (TIF) [file pone.0020271.s001.tif]

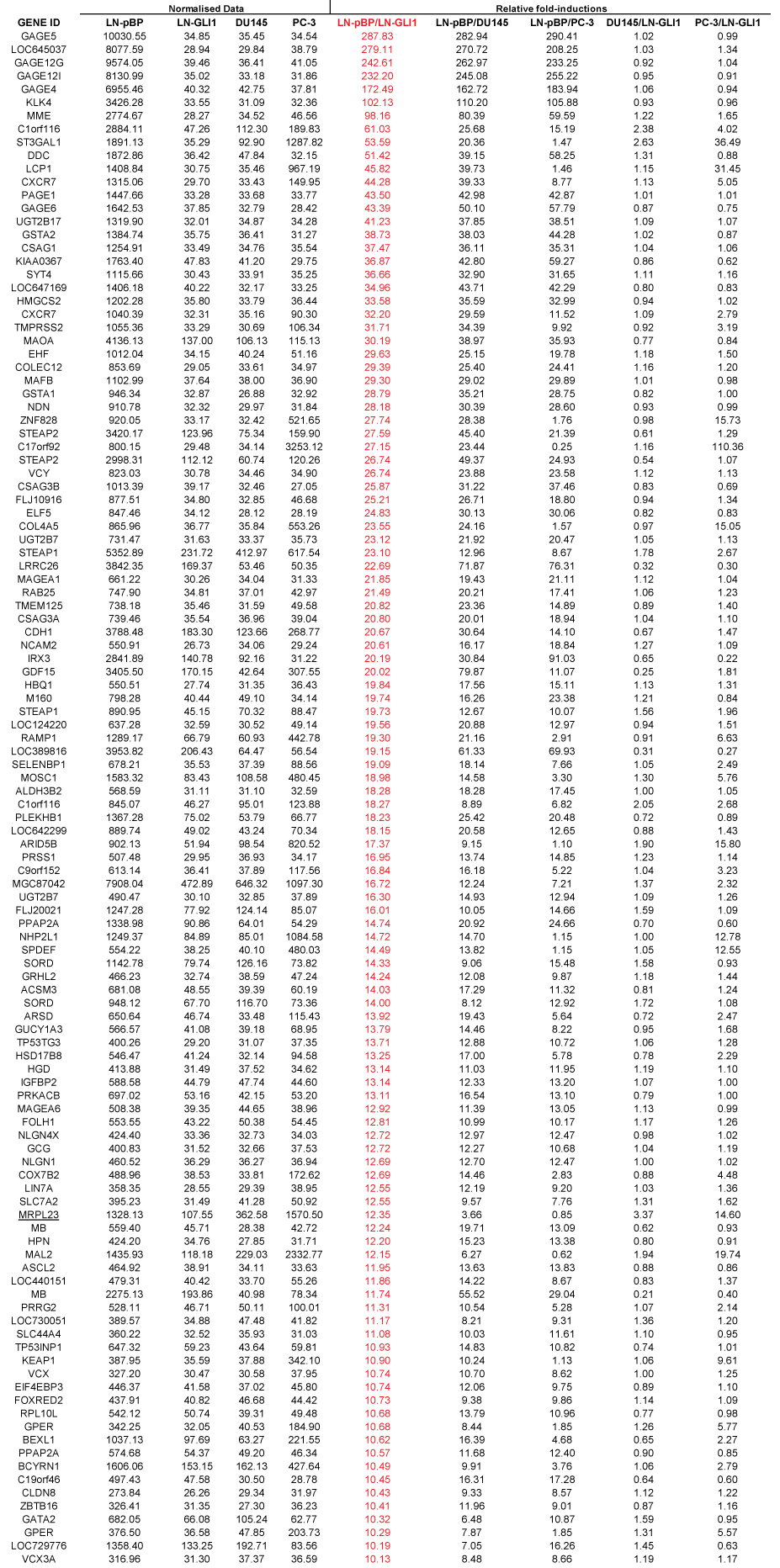

Supplement: Figure S2 — Excel worksheet with the raw expression data of the negatively regulated genes presented within the right heat map of Fig. 3B . (TIF) [file pone.0020271.s002.tif]

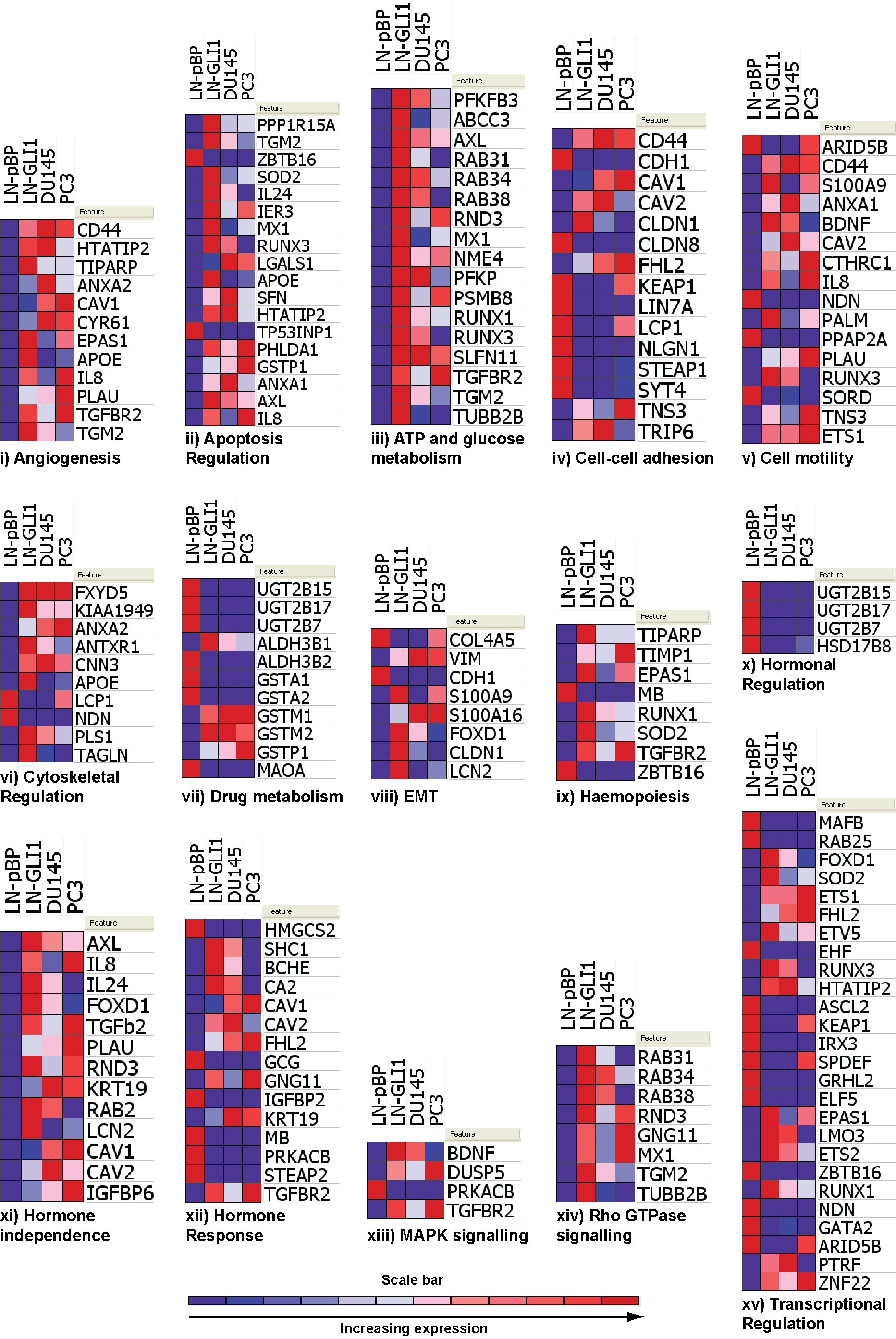

Supplement: Figure S3 — Mini heat maps denoting functional groups of the genes presented in Fig. 3B and Figures S1 and S2. (TIF) [file pone.0020271.s003.tif]

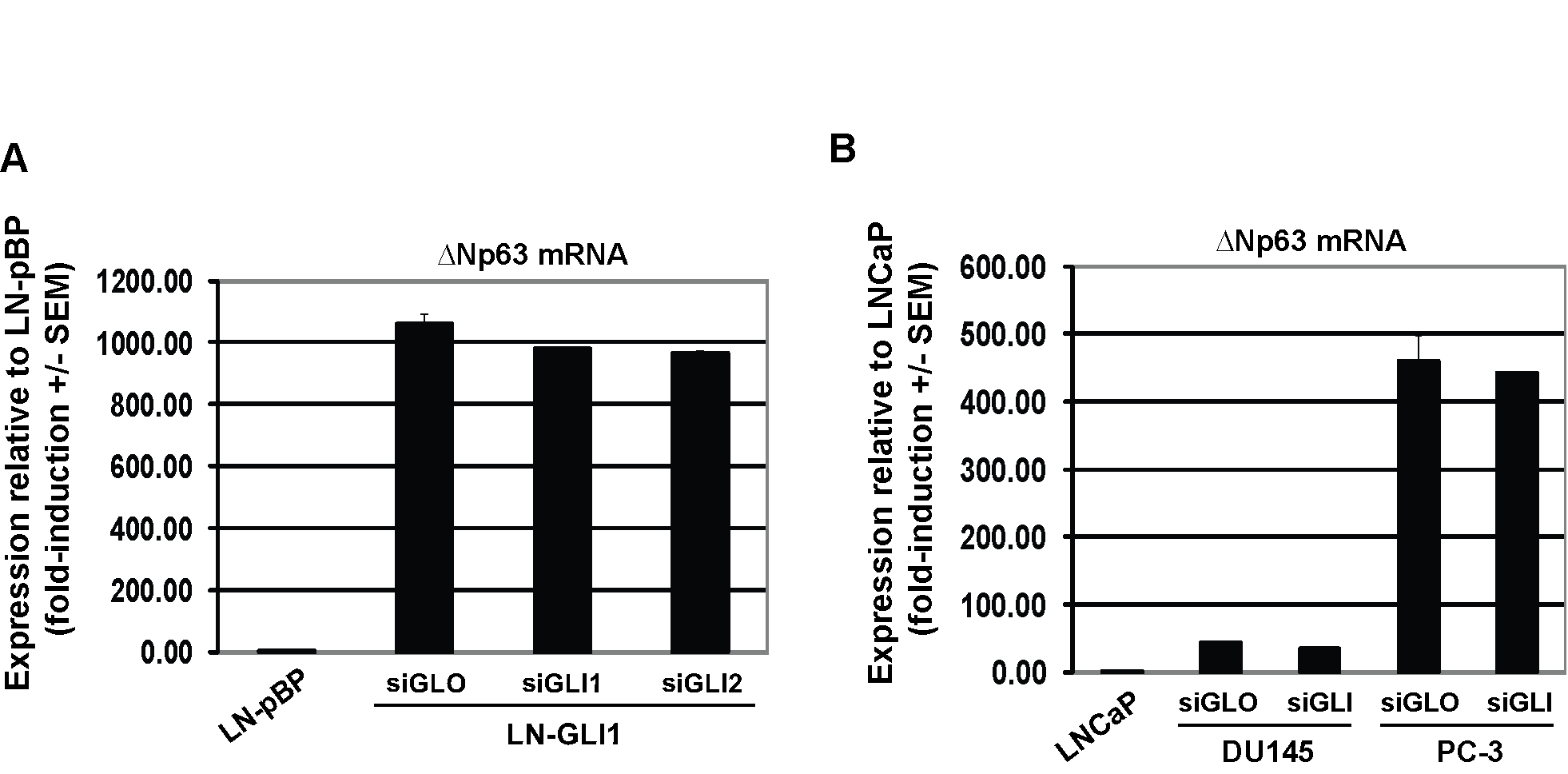

Supplement: Figure S4 — qPCR analysis of ΔNp63 mRNA expression in LNCaP-GLI1, DU145 and PC-3 cells. (TIF) [file pone.0020271.s004.tif]
